# Fructose Induced Endotoxemia in Pediatric Nonalcoholic Fatty Liver Disease

**DOI:** 10.1155/2014/560620

**Published:** 2014-09-28

**Authors:** Ran Jin, Andrew Willment, Shivani S. Patel, Xiaoyan Sun, Ming Song, Yanci O. Mannery, Astrid Kosters, Craig J. McClain, Miriam B. Vos

**Affiliations:** ^1^Department of Pediatrics, School of Medicine, Emory University, 2015 Uppergate Drive NE, Atlanta, GA 30322, USA; ^2^Medical University of South Carolina, Charleston, SC 29425, USA; ^3^Department of Statistics, Emory University, Atlanta, GA 30322, USA; ^4^School of Medicine, University of Louisville, Louisville, KY 40202, USA; ^5^Robley Rex Louisville VAMC, Louisville, KY 40206, USA; ^6^Children's Healthcare of Atlanta, Atlanta, GA 30329, USA

## Abstract

In preclinical studies of fructose-induced NAFLD, endotoxin appears to play an important role. We retrospectively examined samples from three pediatric cohorts (1) to investigate whether endotoxemia is associated with the presence of hepatic steatosis; (2) to evaluate postprandial endotoxin levels in response to fructose beverage in an acute 24-hour feeding challenge, and (3) to determine the change of fasting endotoxin amounts in a 4-week randomized controlled trial comparing fructose to glucose beverages in NAFLD. We found that adolescents with hepatic steatosis had elevated endotoxin levels compared to obese controls and that the endotoxin level correlated with insulin resistance and several inflammatory cytokines. In a 24-hour feeding study, endotoxin levels in NAFLD adolescents increased after fructose beverages (consumed with meals) as compared to healthy children. Similarly, endotoxin was significantly increased after adolescents consumed fructose beverages for 2 weeks and remained high although not significantly at 4 weeks. In conclusion, these data provide support for the concept of low level endotoxemia contributing to pediatric NAFLD and the possible role of fructose in this process. Further studies are needed to determine if manipulation of the microbiome or other methods of endotoxin reduction would be useful as a therapy for pediatric NAFLD.

## 1. Introduction 

Nonalcoholic fatty liver disease (NAFLD) is a leading cause of chronic liver disease and is estimated to affect 40% of obese adolescents in the United States [1]. In adults with NAFLD, circulating endotoxin (lipopolysaccharide or LPS) has been reported to be elevated [2, 3]. Endogenous antibodies against endotoxin are also increased in adults with biopsy-proven nonalcoholic steatohepatitis (NASH), suggesting chronic exposure [4]. However, studies in the pediatric population remain scarce and it is less clear whether or not endotoxin is an important mediator of NAFLD in the early forms of the disease as seen in children. A study by Alisi et al. found increased endotoxin levels among children with NAFLD compared to healthy weight controls [5], but endotoxin could also be associated with obesity* per se *[6, 7], thus warranting further examination.

Animal models have demonstrated that a high-fructose regimen causes increased portal blood endotoxin levels and hepatic steatosis [8, 9]. In mice, reduction of endotoxin using oral antibiotics improved both hepatic steatosis and inflammation [9]. In spite of the growing body of evidence associating fructose with endotoxemia and the metabolic syndrome from preclinical studies, data from human studies on fructose and endotoxin are scarce. In particular, adolescents are an important group to focus on because of their high intake of fructose [10] and the increased prevalence of NAFLD [1].

In the current study, we utilized stored samples from three separate cohorts and sought to answer two distinct questions: (1) are endotoxin levels increased in adolescents with NAFLD (as shown in adults)? (2) Is there a link between fructose consumption and endotoxin levels in adolescents with NAFLD?

## 2. Methods

### 2.1. Subjects and Study Design

This analysis utilized data and samples from a cross-sectional study and two prospective studies of fructose consumption in adolescents with and without NAFLD. The primary outcomes have been previously reported by our research group [11, 12]; however, endotoxin levels have not been evaluated and associated with NAFLD. All studies were approved by the Emory University and Children's Healthcare of Atlanta IRB, and written informed consent (parental consent obtained for subjects <18 years) and assent (when applicable) were obtained for each subject prior to initiation of the study.

Cohort 1 was comprised of 43 Hispanic, obese (BMI *z*-score ≥ 95th percentile for age and gender) adolescents (aged 11–18 years), who had self-reported high consumption of sweet beverages (at least 3 servings of 12 fl oz per day on average). Because both Hispanic ethnicity [13] and high intake of sweet beverages [14] have been reported to be risk factors for hepatic steatosis, we were able to recruit a group of adolescents in this cohort who were likely to have increased risk of significant steatosis but who had not been previously diagnosed or treated for NAFLD. The subjects and exclusion criteria were carefully described in a previously published article [12]. All recruited participants underwent magnetic resonance spectroscopy (MRS) to quantify their hepatic fat. Subjects with hepatic fat > 5% were considered as having steatosis [15] (*n* = 32), or they were classified as obese controls (*n* = 11) if MRS-documented hepatic fat < 5%.

Cohort 2 included a total of 15 adolescents, with 8 being biopsy-proven NAFLD (7 were NASH) and 7 being matched healthy controls. The study design was described elsewhere in detail [11]. Briefly, it was a 24-hour, randomized, crossover feeding study and participants were randomized to either glucose or fructose beverages at visit 1 and the other sugar beverage at visit 2. For each of two visits, subjects arrived at the inpatient research unit after overnight fasting (>12 hours) and consecutively consumed three study-provided standardized meals (containing 50% carbohydrates, 30% fat, and 20% protein) for breakfast (0800 h), lunch (1200 h), and dinner (1600 h), along with either high fructose or glucose (33% of total estimated daily calories) beverages. Their blood samples were drawn at 0800 h (baseline) before feeding, 1 h after breakfast, and, subsequently, every 2 h until the following morning except at 0400 h to allow the adolescents to sleep. Subjects with nonhemolyzed baseline samples and sufficient postprandial samples were included for the analysis of endotoxin.

Cohort 3 was a subgroup of adolescents from cohort 1 who had elevated hepatic fat (>8% by MRS) and consented to participate in a 4-week calorie-matched, randomized, controlled trial. Randomization and study design in detail can be retrieved at clinicaltrials.gov, NCT01188083, and from our previous publication [12]. In brief, subjects were randomly assigned to study-provided fructose or glucose beverages (3 servings of 12 fl oz bottles each day) to replace their usual consumption of sweet beverages. These beverages contained 33 grams of sugar (standard amount of sugar in a typical soda) in the form of either glucose or fructose, matched for color and flavoring (Power Brands, Beverly Hills, CA). Follow-up visits were scheduled at 2 and 4 weeks after the initiation of randomization to monitor body weight and hepatic fat. Fasting blood samples were also collected for the evaluation of metabolic parameters. At the time of the endotoxin study, a total of 16 subjects had sufficient, nonhemolyzed blood samples available from baseline, 2 and 4 weeks.

### 2.2. Measurement of Hepatic Fat

Hepatic fat was assessed by MRS using a rapid 15 sec acquisition technique obtained during a single breath hold [16]. The sequence is constructed from five concatenated echoes using a fixed set of echo times (TE) (12, 24, 36, 48, and 72 ms), with each echo having a repetition time (TR) = 3000 ms, voxel = 3 × 3 × 3 cm^3^, 1024 points, and 1200 Hz bandwidth. Data were exported off-line for automatic processing with in-house software (Matlab, MathWorks, Natick, MA). Water and lipid magnitude spectra were analyzed by determining the area under the curve (AUC) corresponding to a user-defined frequency range surrounding the corresponding water/lipid peaks (water peak: 4.6 ppm; lipid peak: 1.3, 2.0 ppm). The integrated magnitude signals at each TE were fit to exponential *T*2 decay curves, whereby the equilibrium signal (M0) and the relaxation rate (*R*2 = 1/*T*2) were determined by least-squares regression approximation. Using M0 for water and lipid, the *T*2-corrected hepatic lipid fraction was calculated from % hepatic lipid = M0lipid/(M0lipid + M0water).

### 2.3. Laboratory Measurement

Serum alanine aminotransferase (ALT) and aspartate aminotransferase (AST) were examined by the Emory University Hospital clinical laboratory. Plasma glucose, insulin, and high sensitivity C-reactive protein (hs-CRP) were measured with immunoturbidimetric methods (Sekisui Diagnostics, Exton, PA) using AU480 chemistry analyzer (Beckman Coulter), by the Emory Lipid Research Laboratory. Plasma tumor necrosis factor-α (TNF-α) and monocyte chemoattractant protein-1 (MCP-1) were determined with multianalyte chemiluminescence detection using Luminex xMap technology (Millipore Corporation, St. Louis, MO). Endotoxin levels were evaluated by colorimetric assay. Plasma samples were diluted 2-fold in pyrogen-free water, mixed, and heated at 75°C for 10 min to remove nonspecific inhibitors of endotoxin. Samples were allowed to cool to room temperature before the colorimetric assay using the limulus amoebocyte lysate (LAL) kit (Lonza Walkersville, MD). Standards and samples were incubated with LAL for 10 min at 37°C followed by 6 min incubation with colorimetric substrate. The reaction was stopped with 25% acetic acid, and the absorbance was read at 405 nm.

### 2.4. Insulin Resistance Index

Insulin resistance was assessed by the homeostasis model of assessment—insulin resistance (HOMA-IR), which was calculated by glucose (mmol/L) × insulin  (mU/L)/22.5 at fasting state.

### 2.5. Statistical Analyses

Statistical analyses were performed using SAS 9.1. Results in tables were expressed as mean (SD) unless indicated otherwise. Data were examined for normality and equal variance prior to any analyses. Independent two sample *t*-tests or alternatively Mann-Whitney tests (if not normally distributed) were used for comparison between adolescents with and without hepatic steatosis and between adolescents randomized to glucose or fructose beverage groups. Multiple linear regression models were performed to adjust metabolic parameters including BMI *z*-score, HOMA-IR, and hs-CRP. Paired *t*-tests were conducted to determine the significance of percent change in endotoxin levels at weeks 2 and 4, as compared to baseline. The correlations of endotoxin were examined using bivariate correlation tests. In the feeding study, the 9-hour (9-h) and 23-hour (23-h) incremental areas under the curve (IAUC) were calculated for endotoxin by using the trapezoidal method, and independent comparisons were performed for each single time point between NAFLD and non-NAFLD subjects.

## 3. Results

### 3.1. Cross-Sectional Comparison of Fasting Endotoxin Level (Cohort 1)

**Figure 1:**
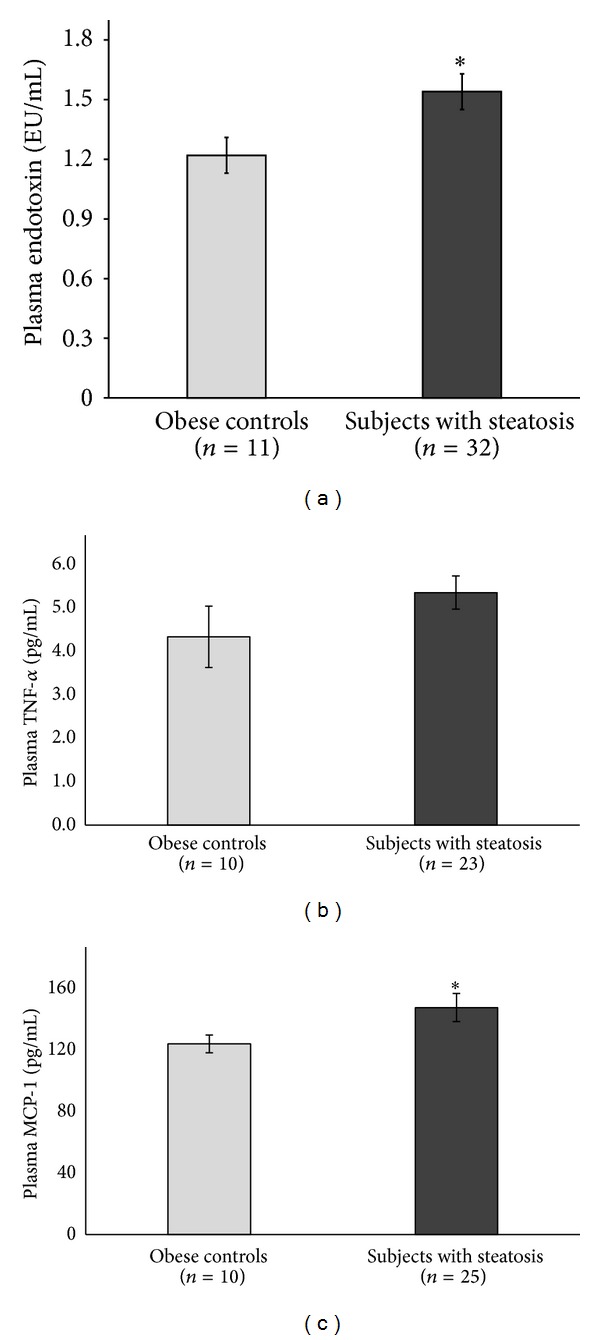
Obese adolescents with hepatic steatosis (>5% by MRS) had increased (a) plasma endotoxin levels, (b) plasma TNF-α levels, and (c) plasma MCP-1 levels as compared to obese adolescents without significant steatosis (hepatic fat < 5% by MRS); *P < 0.05.

Anthropometrics and laboratory parameters for the 32 obese adolescents with hepatic steatosis and the 11 obese adolescents without hepatic steatosis are reported in Table 1. There were no significant differences in age, gender, body weight, BMI *z*-score, and hs-CRP between the two groups. Adolescents with hepatic steatosis (>5% by MRS) had increased ALT (*P* = 0.021), AST (*P* < 0.001), fasting insulin (*P* = 0.010), and insulin resistance as assessed by HOMA-IR (*P* = 0.013) compared with obese control adolescents. The plasma concentration of endotoxin in obese adolescents without hepatic steatosis averaged 1.22 ± 0.30 EU/mL (mean ± SD, ranging from 0.64 to 1.61 EU/mL), while in participants with steatosis, the mean endotoxin level was significantly increased to 1.54 ± 0.52 EU/mL (mean ± SD, ranging from 0.85 to 2.83 EU/mL) (*P* = 0.019) (Figure 1(a)). In multiple linear regression models, we found that the difference in endotoxin levels between subjects with and without steatosis remained significant after adjusting for BMI *z*-score (*P* = 0.036) and for hs-CRP (*P* = 0.042), respectively, but was blunted after the adjustment for HOMA-IR (*P* = 0.068) and for the cluster of HOMA-IR, BMI *z*-score, and hs-CRP (*P* = 0.056).

We further examined TNF-α and MCP-1 levels on a subgroup of subjects who had sufficient blood samples available. We found that adolescents with steatosis had a trend towards higher TNF-α (mean ± SD: 5.46 ± 1.89 versus 4.42 ± 2.28 pg/mL, *P* = 0.18) and significantly increased MCP-1 (mean ± SD: 150 ± 46.4 versus 126 ± 18.5 pg/mL, *P* = 0.034) compared to their obese controls (Figures 1(b) and 1(c)) and that plasma endotoxin amount was positively correlated with TNF-α (*r* = 0.471, *P* = 0.006), MCP-1 (*r* = 0.337, *P* = 0.047), and HOMA-IR (*r* = 0.381, *P* = 0.013).

### 3.2. Endotoxin Response to Acute Feeding Challenge (Cohort 2)

**Figure 2:**
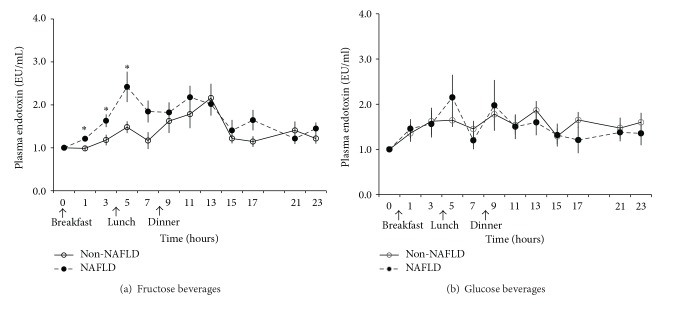
Postprandial plasma endotoxin levels in response to (a) fructose and (b) glucose beverages given with breakfast, lunch, and dinner. The solid line represents 7 children without NAFLD and the dashed line shows the response in 8 children with biopsy-proven NAFLD. Baseline values were set as reference (1.0) and the following time points represent the ratio to baseline. *P < 0.05 when comparing NAFLD and non-NAFLD subjects at given time point.

Next, we evaluated postprandial endotoxin levels in samples from a group of 15 adolescents, 8 of whom had histologically confirmed NAFLD and 7 matched healthy adolescents. Their baseline characteristics are summarized in Table 2. In response to fructose beverages (consumed with meals), adolescents with NAFLD had an acute increase of plasma endotoxin levels after 1, 3, and 5 hours (*P* < 0.05 for all) comparing to non-NAFLD subjects. This resulted in an elevation of 9-h IAUC of postprandial endotoxin in NAFLD compared to their healthy controls (mean ± SE: 6.85 ± 1.49 versus 2.50 ± 0.87, *P* = 0.026). The biggest differences were seen after breakfast and lunch and less variation was seen overnight; thus their 23-h IAUC comparison (mean ± SE: 15.11 ± 3.83 versus 10.19 ± 4.23, *P* = 0.23) did not reach statistical significance (Figure 2(a)). In contrast, no significant difference of postprandial endotoxin in response to glucose beverages (consumed with meals) was observed between adolescents with and without NAFLD (Figure 2(b)).

### 3.3. Endotoxin Response to 4-Week Beverage Trial (Cohort 3)

**Figure 3:**
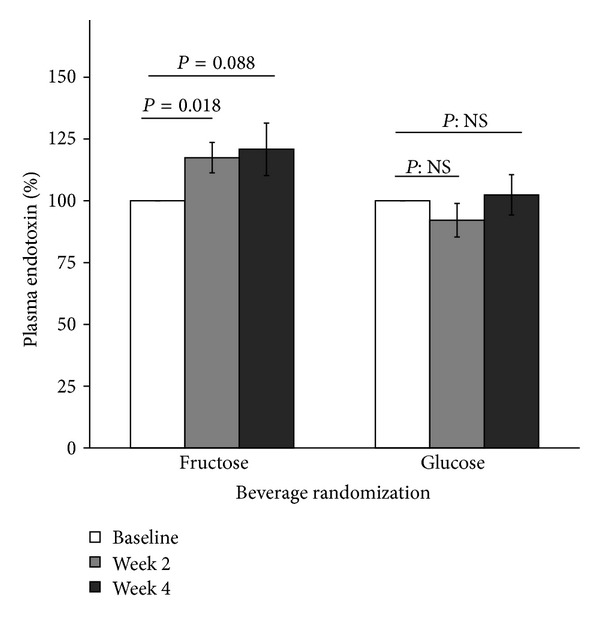
Percentage change of plasma endotoxin level in adolescents with NAFLD after 2- and 4-week ingestion of study-provided fructose or glucose-only beverages. Baseline values were set as reference (100%). Error bars stand for SE.

Finally, we measured endotoxin in samples from adolescents with NAFLD who participated in a 4-week study of fructose beverages compared to glucose beverages. The baseline characteristics of the 16 adolescents with hepatic steatosis who participated in the 4-week beverage trial are presented in Table 3. There were no significant differences in age, gender, weight, glycemic status, and lipid profile between the two groups. Compared to baseline, after drinking 3 study-provided fructose beverages per day, participants had significantly increased fasting plasma endotoxin levels at 2 weeks (mean ± SD: 1.21 ± 0.29 versus 1.45 ± 0.50 EU/mL, *P* = 0.018) and a trend for increased endotoxin at 4 weeks (mean ± SD: 1.21 ± 0.29 versus 1.47 ± 0.53 EU/mL, *P* = 0.088), while adolescents who consumed glucose beverages did not have increased endotoxin levels (mean ± SD: 1.61 ± 0.69, 1.39 ± 0.38, and 1.55 ± 0.55 EU/mL at weeks 0, 2, and 4, resp.) (Figure 3).

## 4. Discussion

Children and adolescents are an important group in which one can study the mechanisms leading to NAFLD because they have an early, possibly more aggressive form of the disease [17]. In addition, they are less likely to have other chronic diseases which could alter gut permeability as well as endotoxin transfer and/or clearance. Through these analyses, we found that obese adolescents with elevated hepatic steatosis had increased plasma endotoxin levels compared to those with normal hepatic fat (<5% by MRS) even after multiple adjustments for metabolic markers, suggesting a possible role for endotoxin in the mechanism of pediatric NAFLD. Second, we observed that adolescents with NAFLD (mostly NASH) had elevated postprandial endotoxin compared to healthy individuals. Finally, in a 4-week randomized controlled trial, we found a trend for increased fasting plasma endotoxin after exposure to fructose drinks.

A previous study by Alisi et al. suggested that endotoxin levels were increased in pediatric NAFLD compared to healthy weight subjects [5]. We compared endotoxin levels in a weight and ethnicity matched cohort because obesity has been indicated to be associated with dysfunction/disturbance of the gastrointestinal barrier and consequently increased entry of endotoxin into the circulation [18, 19]. It has also been shown that diabetic patients appear to have higher endotoxin amounts suggesting a correlation between endotoxin and insulin resistance [20]. In the current study, by adjusting for BMI, insulin resistance, and inflammatory marker hs-CRP, we found that the elevation of endotoxin appears to be strongly related to the presence of hepatic steatosis. Both endotoxin and its resultant inflammatory perturbation appear to trigger hepatic and peripheral insulin resistance [21]. Agwunobi et al. published data using euglycemic clamps that demonstrated hepatic insulin resistance following LPS administration [22], and Mehta et al. reported a 35% decrease in insulin sensitivity induced by endotoxemia [23]. Cytokines such as TNF-α can inhibit insulin receptor signaling and target insulin receptor substrate proteins for degradation [24]. Furthermore, endotoxin is also known to markedly induce MCP-1 [25] which can recruit the C-C motif chemokine receptor-2 (CCR2)-expressing monocytes in adipose tissue, and CCR2 associates adipose tissue inflammation and systemic insulin resistance [26]. However, given the fact that NAFLD (particularly in its advanced form NASH) has a nearly universal interrelationship with obesity, insulin resistance, and inflammation, it is difficult to distinguish between the effects of endotoxemia on hepatic fat (NAFLD) and its coexisting metabolic morbidities.

The idea that fructose induced endotoxin might contribute to NAFLD has been studied in animal models and adults, but little data were previously available in children. In a fructose fed mouse model, antibiotics reduced fructose induced endotoxin in the portal blood and improved hepatic steatosis and inflammation [9]. Subsequent studies demonstrated that endotoxin stimulates the innate immune system via toll-like receptor 4 (TLR4) to increase inflammation in the liver [8]. In mechanism studies, increased endotoxin release from the gut can activate TLR4 to stimulate myeloid differentiation factor 88 (MyD88) [27]. This interaction of TLR4 and MyD88 triggers the downstream signaling cascade leading to the activation of the nuclear factor *κ*B (NF-*κ*B) pathway, further releasing inflammatory cytokines such as TNF-α and IL-6, which, in turn, can result in liver injury [27]. Furthermore, animals with NAFLD have been shown to have partial loss of the tight junction protein, occluden-1, contributing to increased intestinal permeability and translocation of intestinal endotoxin [28]. Fructose could make this worse because tight junction proteins have been shown to be markedly lower in mice chronically exposed to fructose in comparison to water-fed controls [29]. Supporting this, mice fed with significantly greater amounts of fructose had elevated plasma endotoxin levels and higher hepatic expression of genes of the TLR-4-dependent signaling cascade [30].

To evaluate the above effects in pediatric NAFLD, we studied endotoxin levels in two situations: after acute consumption of fructose and after weeks of fructose beverages. Both NAFLD and non-NAFLD subjects had an acute elevation of postprandial endotoxin concentrations after fructose beverages (consumed with meals) in the 24-hour acute feeding challenge, but there was a significantly higher response observed in adolescents with NAFLD. This increased susceptibility in NAFLD might be explained by increased gut permeability, disruption of intestinal tight junctions, and possible alterations in the microbiome as indicated in adults [28, 31]. A recent study by Giorgio et al. further reported increased intestinal permeability in children with NAFLD [32]. Alternatively, the increased endotoxin could be due to impaired Kupffer cell function. Normally, endotoxin released from the gut is cleared rapidly on first pass by Kupffer cells to prevent its escape into the systemic circulation. In NAFLD, this hepatic clearance may be disturbed and insufficient [33] and could be responsible for the increased level of circulating endotoxin.

Because we saw postprandial increases in the acute feeding challenge study, we evaluated endotoxin in a longer, calorie-matched, randomized controlled beverage trial in adolescents with hepatic steatosis comparing fructose beverages to glucose beverages. We found that 4 weeks of glucose beverages did not further exacerbate endotoxin levels, while continuous provision of fructose beverages resulted in elevation of endotoxin after just 2 weeks with a trend towards an increase at 4 weeks (no longer significant because of subject variability). A pilot study in adults also showed a decrease in endotoxin in response to fructose reduction, although over 6 months. In that study, subjects with NAFLD who consumed 50% less fructose compared to baseline had significantly lower levels of endotoxin as well as hepatic lipid contents [34]. However, causality could not be proven because the subjects also lost weight. In our trial, the body weights remained stable from baseline to the end of the intervention. Putting our data together with the previous studies, it appears possible that when subjects are chronically exposed to a high fructose environment, endotoxemia and subsequent activation of inflammatory cytokines occur and promote insulin resistance in the liver thus contributing to NAFLD.

Strengths of our studies include (1) the well-matched cohorts allowing us to isolate the effect of NAFLD and examine its associations; (2) the precise measurement of hepatic steatosis by MRS; and (3) the utilization of an inpatient feeding study methodology conducted in a metabolic unit. Importantly, in both cohorts 2 and 3, the experiments were randomized, controlled tests of fructose administration in comparison to a calorically matched beverage (glucose drinks). This methodology provides the strongest evidence that fructose is possibly causing the differences seen.

There were several limitations in these studies. Our subjects in cohort 1 are Hispanic-American adolescents; thus our findings may not be generalizable to other racial and ethnic groups, particularly African-American children, who develop NAFLD less often. In our acute feeding challenge study (cohort 2), endotoxemia peaked rapidly after meals and normalized overnight. Because we measured blood every two hours, we may have missed the true peak level(s) of endotoxin. Further, we were unable to determine if endotoxin was elevated because of slower clearance or increased translocation into the bloodstream and whether this increase of endotoxin was caused by the presence of hepatic steatosis or its closely associated metabolic perturbations. In the 4-week randomized controlled trial (cohort 3), it is possible that participants made other changes to their diets in response to the research beverages even though they were requested to keep their diet pattern. Finally, the study did not include stool so we were unable to assess if the endotoxin changes seen were secondary to a change in the microbiota.

In conclusion, we demonstrated that exposure to high fructose both acutely (postprandial) and chronically (2 weeks) is associated with an increase of circulating endotoxin in adolescents with NAFLD, and we also demonstrated correlations of endotoxin with markers of insulin resistance and inflammation. Fructose reduction is a feasible side-effect-free strategy for patients with NAFLD to prevent disease progression. However, further studies will be necessary to prove the therapeutic and possibly preventive benefits of fructose reduction.

## Figures and Tables

**Table 1:** Anthropometrics and laboratory parameters of the 32 adolescents with hepatic steatosis and the 11 obese controls at fasting state: study cohort 1.

Parameters, mean (SD)	Obese controls	Subjects with steatosis	*P* value
	(<5% by MRS, *n* = 11)	(>5% by MRS, *n* = 32)	
Age, years	14.3 (1.85)	13.7 (2.65)	0.443
Male (*n*, %)	4 (36.4)	13 (40.6)	0.803
Weight (kg)	82.9 (21.5)	80.5 (14.9)	0.967
BMI *z*-score	2.00 (0.22)	2.17 (0.37)	0.129
ALT (U/L)	17.8 (7.60)	49.7 (89.5)	0.021
AST (U/L)	21.9 (3.99)	68.2 (180)	<0.001
Hepatic fat (%)	3.87 (0.62)	11.2 (5.27)	<0.001
Glucose (mmol/L)	5.13 (0.89)	5.18 (0.90)	0.978
Insulin (mU/L)	18.2 (6.69)	36.2 (30.5)	0.010
HOMA-IR	4.06 (1.31)	8.79 (9.13)	0.013
hs-CRP (mg/L)	3.17 (3.44)	4.98 (5.92)	0.278

BMI: body mass index; ALT: alanine aminotransferase; AST: aspartate aminotransferase; HOMA-IR: homeostatic model assessment for insulin resistance, calculated as fasting glucose (mmol/L) × insulin (mU/L)/22.5; hs-CRP: high sensitivity C-reactive protein.

**Table 2:** Baseline characteristics of the 8 adolescents with biopsy-proven NAFLD and the 7 healthy controls: study cohort 2.

Parameters, mean (SD)	non-NAFLD (*n* = 7)	NAFLD (*n* = 8)	*P* value
Age, years	13.7 (2.22)	13.0 (2.73)	0.315
Male, *n* (%)	5 (71.4)	8 (100)	0.104
BMI *z*-score	0.18 (0.65)	2.29 (0.38)	0.001
Hepatic fat, %	1.02 (1.18)	22.0 (6.16)	0.001
ALT (U/L)	14.6 (2.51)	130 (63.2)	0.001
AST (U/L)	23.4 (4.30)	79.6 (40.6)	0.001
Glucose (mmol/L)	5.53 (0.40)	5.32 (1.06)	0.487
Insulin (mU/L)	9.67 (12.4)	42.7 (27.7)	0.005
HOMA-IR	2.27 (2.70)	10.2 (6.99)	0.016
hs-CRP (mg/L)	0.25 (0.49)	2.95 (3.33)	0.004

BMI: body mass index; ALT: alanine aminotransferase; AST: aspartate aminotransferase; HOMA-IR: homeostatic model assessment for insulin resistance, calculated as fasting glucose (mmol/L) × insulin (mU/L)/22.5; hs-CRP: high sensitivity C-reactive protein.

**Table 3:** Baseline characteristics of participants enrolled in the 4-week beverage trial: study cohort 3.

Parameters, mean (SD)	Fructose (*n* = 8)	Glucose (*n* = 8)	*P* value
Age (years)	14.6 (2.50)	13.3 (2.32)	0.273
Male, *n* (%)	3 (37.5)	4 (50.0)	0.614
Body weight (kg)	86.1 (13.3)	81.3 (15.9)	0.521
BMI *z*-score	2.32 (0.56)	2.01 (0.26)	0.184
Hepatic fat (%)	14.5 (5.73)	12.1 (4.82)	0.382
ALT (U/L)	35.1 (20.5)	31.3 (18.6)	0.698
AST (U/L)	32.1 (9.76)	32.9 (7.45)	0.865
Triglycerides (mmol/L)	161 (111)	175 (58.5)	0.768
Cholesterol (mmol/L)	166 (28.8)	170 (48.8)	0.836
LDL (mmol/L)	106 (32.5)	105 (38.6)	0.967
HDL (mmol/L)	45.1 (9.84)	45.0 (9.83)	0.986
FFA (mmol/L)	0.97 (0.24)	1.16 (0.51)	0.342
Glucose (mmol/L)	5.46 (0.85)	4.97 (1.59)	0.461
Insulin (mU/L)	30.0 (13.7)	31.4 (30.5)	0.906
HOMA-IR	7.17 (3.03)	7.23 (8.28)	0.985
hs-CRP (mg/L)	4.22 (3.03)	3.16 (2.54)	0.242

ALT: alanine aminotransferase; AST: aspartate aminotransferase; LDL: low-density lipoprotein; HDL: high-density lipoprotein; FFA: free fatty acid; HOMA-IR: homeostatic model assessment for insulin resistance index, calculated as fasting glucose (mg/dL) × insulin (*µ*U/L)/405; hs-CRP: high sensitivity C-reactive protein.
